# GC-1 mRHBDD1 knockdown spermatogonia cells lose their spermatogenic capacity in mouse seminiferous tubules

**DOI:** 10.1186/1471-2121-10-25

**Published:** 2009-04-10

**Authors:** Yong Wang, Wei Song, Shuchun Li, Xin Guan, Shiying Miao, Shudong Zong, SS Koide, Linfang Wang

**Affiliations:** 1 State Key Laboratory of Medical Molecular Biology, Institute of Basic Medical Sciences, Chinese Academy of Medical Sciences, Peking Union Medical College, Tsinghua University 5 Dong Dan San Tiao, Beijing 100005, PR China; 2 National Research Institute for Family Planning Beijing, WHO Collaboration Center of Human Reproduction 12 Da Hui Si, Hai Dian, Beijing 100081, PR China; 3 Center for Biomedical Research, the Population Council, 1230 York Avenue, New York, NY 10021, USA

## Abstract

**Background:**

Apoptosis is important for regulating spermatogenesis. The protein mRHBDD1 (mouse homolog of human RHBDD1)/rRHBDD1 (rat homolog of human RHBDD1) is highly expressed in the testis and is involved in apoptosis of spermatogonia. GC-1, a spermatogonia cell line, has the capacity to differentiate into spermatids within the seminiferous tubules. We constructed mRHBDD1 knockdown GC-1 cells and evaluated their capacity to differentiate into spermatids in mouse seminiferous tubules.

**Results:**

Stable mRHBDD1 knockdown GC-1 cells were sensitive to apoptotic stimuli, PS341 and UV irradiation. *In vitro*, they survived and proliferated normally. However, they lost the ability to survive and differentiate in mouse seminiferous tubules.

**Conclusion:**

Our findings suggest that mRHBDD1 may be associated with mammalian spermatogenesis.

## Background

Spermatogenesis generates functional sperm cells from initially undifferentiated germ cells. This involves the proliferation of spermatogonia, meiosis of spermatocytes and the differentiation of spermatids into spermatozoa. It is a complex developmental program in which myriad events take place to ensure that the germ cells reach their proper stages of development at the appropriate times. Normal spermatogenesis requires a well-regulated balance of several processes, including cell proliferation, differentiation and apoptosis.

Apoptosis is a key phenomenon during spermatogenesis. For instance, an early, massive wave of germ cell apoptosis occurs at puberty. This event takes place during postnatal weeks 2 to 4 in mice, with a peak after 3 weeks [1-3]. It is estimated that 75% of spermatogenic cells undergo apoptosis during development [4,5], ensuring the maintenance of a critical ratio between maturing germ cells and Sertoli cells [2,6]. Sporadic apoptosis also occurs, primarily in spermatogonia and spermatocytes [2], eliminating defective germ cells with mutated DNA [7].

The Rhomboid family comprises polytopic membrane proteins, which may be the most widely-conserved membrane proteins identified to date [8]. They share conserved biochemical properties in all biological kingdoms. Rhomboid proteases, which have been well studied in *Drosophila*, appear to regulate EGF receptor signalling pathways, thereby controlling growth and development [9,10]. In addition, some yeast Rhomboid proteases have been found to play important roles in mitochondrial membrane remodelling [11], while some parasite proteases containing a Rhomboid domain are important for invasiveness [12].

Vertebrate rhomboid genes have been grouped into three classes: (1) active cellular rhomboids, including RHBDL1, RHBDL2, RHBDL4 and RHBDD1 [13]; (2) inactive cellular rhomboids, including RHBDL5 and RHBDL6; (3) a mitochondrial rhomboid, PARL [14]. However, the physiological functions of these proteases remain to be clarified. Recently, RHBDL2 was found to cleave thrombomodulin and ephrin B3 [15,16]. An exciting finding in this field was the discovery of PARL (Presenilin-associated rhomboid-like), the function of which appears to be associated with apoptosis [17,18]. *Drosophila *Rhomboid 7 is believed to be associated with spermatogenesis, as male mutants tend to be afflicted with familial sterility [19].

mRHBDD1 is the first Rhomboid serine protease known to be involved in mammalian spermatogenesis. We found that RHBDD1, which contains a Rhomboid domain, is highly expressed in testes and is involved in regulating apoptosis [13]. In the present study, the roles of mRHBDD1 in germ cell apoptosis and mouse spermatogenesis were investigated. We found that it is involved in regulating apoptosis in a mouse spermatagonia cell line, GC-1, and is essential for their capacity to differentiate into spermatids in mouse seminiferous tubules.

## Results

### *mrhbdd1 *and *rrhbdd1 *are polytopic genes that are highly expressed in testis

**Figure 1:**
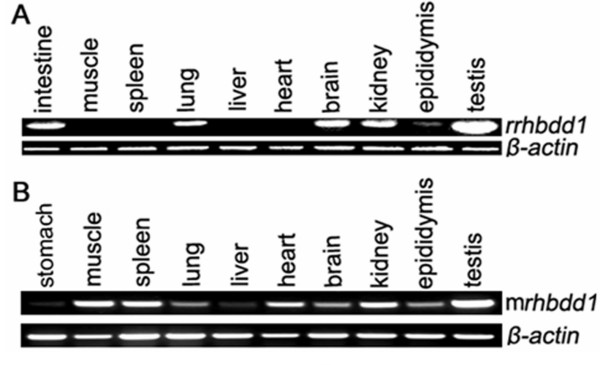
**Expression of *mrhbdd1 *and *rrhbdd1 *in different mouse and rat tissues**. A. *rrhbdd1*, *rhbdd1 *rat homolog, was detected in intestine, muscle, spleen, lung, liver, heart, brain, kidney, epididymis and testis. B. *mrhbdd1, rhbdd1 *mouse homolog, was detected in stomach, muscle, spleen, lung, liver, heart, brain, kidney, epididymis and testis. Note the highest contents in the testes of both animals. Results were obtained using RT-PCR with *β-actin *as the loading control.

*mrhbdd1 *and *rrhbdd1 *were expressed in a variety of mouse and rat tissues, and the highest levels of gene products were found in the testis (Figure 1A and 1B). Previously, a human homolog, *rhbdd1*, was also shown to be highly expressed in testes [13]. These findings suggest that this Rhomboid-containing protein may be involved in regulating mammalian spermatogenesis.

### Apoptotic regulation of mRHBDD1 in a mouse spermatogonia line

**Figure 2:**
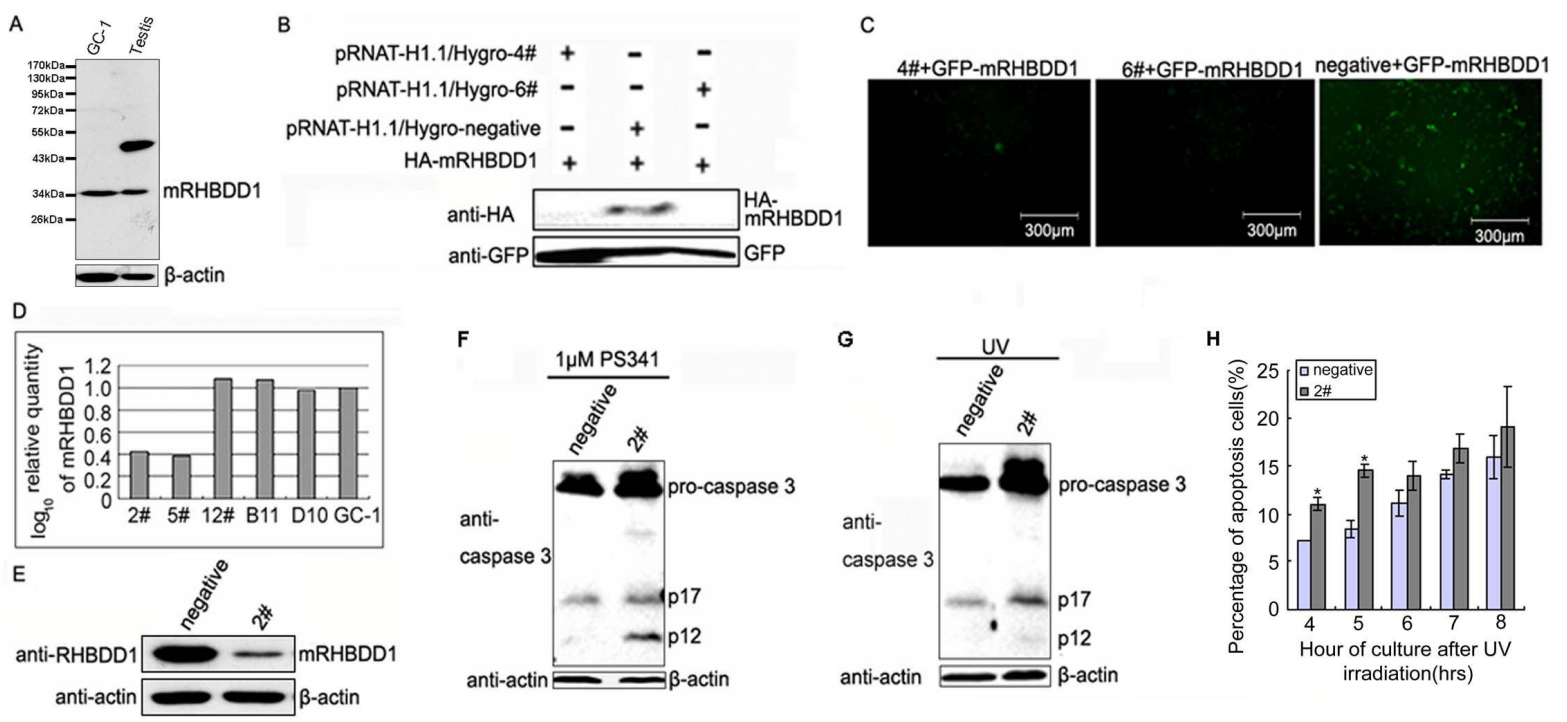
**Apoptosis in mRHBDD1 knock-down GC-1 cells**. A. mRHBDD1 protein levels in GC-1 cells and mouse testis were examined by Western blotting; the same blot was immunoblotted with anti-β-actin antibody as internal control. Protein (30 μg) was separated on a 12% polyacrylamide gel as described in the Methods section. B and C. Screening for effective RNAi plasmids against mRHBDD1. Western blotting indicated that pRNAT-H1.1/Hygro-4# and 6# were both candidates; pRNAT-H1.1/Hygro-6# was selected. GFP was a loading control. D and E. Identification of stable mRHBDD1 knockdown GC-1 cell clones by Real-Time PCR and Western blotting. Clone 2# was selected for the following experiments. F and G. Western blot analysis of caspase 3 cleavage in 2# and negative control GC-1 cells after PS341 or UV treatment. Control cells were subcultured from a selected stable GC-1 cell clone expressing a control negative shRNA. H. FACS assay to analyse apoptosis in 2# and negative control GC-1 cells after UV irradiation. The percentage of apoptotic cells (% of total cells) was determined using the program EXPO32-ADC and shown as a bar chart. * (p < 0.05)

In order to investigate this putative role of mRHBDD1 in spermatogenesis, we studied its expression in a mouse-derived spermatogonia cell line, GC-1 spg (ATCC number: CRL-2053; Figure 2A). Stable mRHBDD1 knockdown shRNA and negative control shRNA GC-1 cells were constructed (Figure 2B–E). Because RHBDD1 regulates apoptosis in some somatic cells [13], the occurrence of apoptosis in mRHBDD1 knockdown GC-1 cells was determined after challenges with apoptotic stimuli, PS341 and UV irradiation. Procaspase 3 cleavage was indicated by an increase in p17 or p12, its cleavage products during apoptosis. As shown in Figure 2F and 2G, these apoptosis markers were more prominent in the mRHBDD1 knockdown GC-1 cells than in the negative control GC-1 cells after exposure to apoptosis inducers, PS341 and UV irradiation. We also confirmed by FACS analysis that apoptosis increased in stable mRHBDD1 knockdown GC-1 cells after UV stimulation (Figure 2H). These results indicate that mRHBDD1 normally inhibits apoptotic activity in spermatogonia.

### Endogenous mRHBDD1 knockdown in GC-1 cells does not affect their proliferation

**Figure 3:**
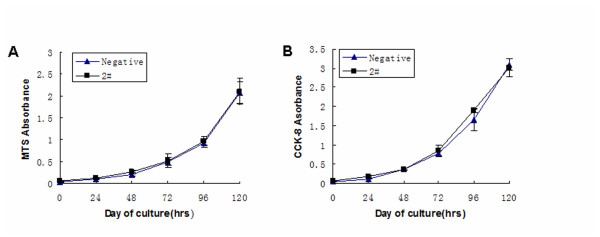
**Proliferation of mRHBDD1 knockdown GC-1 cells and negative control cells**. GC-1/2# cells and GC-1/negative cells were seeded at a density of 1000/well in 96-well plates in DMEM with 10% FBS. Measurements of absorbance at 490 nm for the MTS assay (A) and at 450 nm for the CCK-8 assay (B) were made once per day for 5 days. Results are the means ± SD of three independent experiments.

To investigate the proliferation of mRHBDD1 knockdown GC-1 cells, MTS and CCK-8 assays were performed. Proliferation did not differ between these cells and the negative control cells (Figure 3A and 3B). Thus, mRHBDD1 knockdown GC-1 cells possess normal survival and proliferation capacities under *in vitro *conditions.

### Stable mRHBDD1 knock-down GC-1 cells lose survival and differentiation capacities in mouse seminiferous tubules

**Figure 4:**
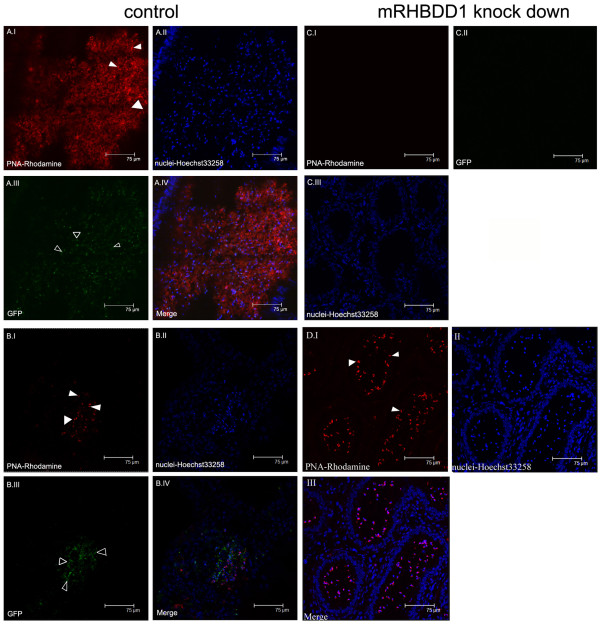
**Confocal images of mRHBDD1 knockdown and control mouse testis sections**. A. Autologous control tubules from the right epididymis of one mouse. pRNAT-H1.1/Hygro-negative control (encoding GFP) stably-expressing GC-1 cells were transplanted into the seminiferous tubules of the right testis. These cells differentiated into spermatids with GFP in the epididymal tubules. They had acrosomes that were stained with PNA-linked Rhodamine. I. PNA-stained acrosomes. II. Hoechst 33258-stained nuclei. III. GFP-labelled spermatids. IV. Merge. B. Heterologous control (seminiferous tubule). Stable pRNAT-H1.1/Hygro-negative control (encoding GFP) GC-1 cells were transplanted into the seminiferous tubules of testes from another animal, capable of producing spermatids in the seminiferous tubules, and were tagged with GFP. They contained acrosome structures stained with PNA-linked Rhodamine. I. PNA-stained acrosomes. II. Hoechst 33258-stained nuclei. III. GFP-labelled spermatids. IV. Merge. C. Test group tubules from the left epididymis of the same animal as in A. pRNAT-H1.1/Hygro-6# containing an effective RNAi plasmid against stable mRHBDD1-expressing GC-1 cells (cell line 2#) was transplanted into the seminiferous tubules of the left testis. The transplanted GC-1 cells and spermatids were not found in the seminiferous or epididymal tubules, indicating that these stable germ cells had died or lacked the ability to differentiate. I. PNA-stained acrosomes. II. GFP-labelled spermatids. III. Hoechst 33258-stained nuclei. D. Residual endogenous germ cells that had resisted busulfan treatment in mouse testes differentiated into sperm cells in the epididymal tubules of the left testis, indicating that these busulfan-treated seminiferous tubules could still support the generation of spermatids. Note: PNA specifically stains the acrosomes of spermatids. I. PNA-stained acrosomes. II. Hoechst 33258-stained nuclei. III. Merge. White triangles are PNA-stained acrosomes (AI, BI and DI). Open triangles are spermatids with GFP tag derived from stable GC-1 cells (AIII, BIII).

The above experiments demonstrating the anti-apoptotic activity of mRHBDD1 in GC-1 cells suggested that this factor is associated with the survival of spermatogonia. To investigate an *in vivo *role for mRHBDD1, mRHBDD1 knockdown GC-1 cells and negative control GC-1 cells were transplanted into mouse seminiferous tubules and their survival and differentiation into spermatids were studied. In this *in vivo *model, mRHBDD1 knockdown GC-1 cells with pRNAT-H1.1/Hygro-6# and negative control cells with pRNAT-H1.1/Hygro-negative (both GFP positive) were transplanted into the seminiferous tubules of, respectively, right and left mouse testes, from which most of the endogenous germ cells had been eliminated by busulfan treatment. As shown in Figure 4A and 4B, autologous and heterologous transplanted control cells, tagged by GFP, differentiated into spermatids, as shown by the presence of acrosomes (stained red by PNA-labelled Rhodamine). However, mRHBDD1-suppressed GC-1 cells did not survive or differentiate into spermatids, as shown by the absence of GFP and acrosomes from the epididymal tubules (Figure 4C). In contrast, the mouse residual germ cells (no GFP tag) that resisted busulfan treatment did differentiate into sperm cells, as shown by the presence of PNA-Rhodamine-labelled acrosomes (Figure 4D). Thus, mRHBDD1 knockdown apparently influences the survival of germ cells and their differentiation into sperm cells *in vivo*.

## Discussion

It is well established that the regulation of apoptosis is a key control mechanism for spermatogenesis [2,6]. An early wave of germinal cell apoptosis during the first round of spermatogenesis in the immature testis is probably a critical requirement during testis development because it maintains a balanced ratio of germ cells to Sertoli cells [20]. Moreover, the elimination of supra-optimal production of spermatogonia by apoptosis is common in the adult testis, where it is primarily restricted to spermatogonia [21]. However, apoptosis of defective germ cells is also essential for maintaining genetic fidelity in the development and survival of offspring. RHBDD1/mRHBDD1 may play a role in regulating these events.

The testis cell transplantation method has been developed as a powerful approach for studying spermatogenesis. It is also used to examine spermatogenic defects, to correct male infertility and to generate transgenic animals [22]. Primary germ cells are the best model to perform these experiments, but there are some puzzles: 1) It is difficult to obtain enough spermatogonia cells from seminiferous tubules since these cells are a minority among germ cells. 2) The transfection efficiency of pRNAT-H1.1/Hygro-6# into spermatogonia is very low. 3) Most of spermatogonia transfected by plasmids will undergo death. To resolve the above problems, we have established a method for studying mammalian spermatogenesis using a GC-1 spermatogonia line transplantation model. Its distinct advantage is to make it much easier to obtain highly-purified GC-1 spermatogonia cells for experiments than by purifying primary spermatogonia cells from mouse seminiferous tubules.

The results of the current study show that transplanted control GC-1 cells can differentiate from spermatogonia into spermatids, indicating that they possess the properties of germ stem cells, capable of differentiating in an *in vivo *microenvironment. Furthermore, the appearance of spermatids derived from control GC-1 cells and from the residual endogenous germ cells that had resisted busulfan treatment in the seminiferous tubules showed that these damaged tubules maintained the ability to generate spermatids from spermatogonia. Thus, the disappearance of the mRHBDD1 knockdown GC-1 cells from the seminiferous tubules 8 weeks after transplantation is the direct result of endogenous mRHBDD1 suppression.

mRHBDD1 has anti-apoptotic potential, and its suppression in GC-1 cells vitiates spermatogonia survival and differentiation *in vivo*. The mRHBDD1 knockdown GC-1 cells did not disappear from the mouse seminiferous tubules because they entered a morbid state and died; they showed normal survival and differentiation *in vitro *like the negative control GC-1 cells. Although the mechanism by which mRHBDD1 regulates mammalian spermatogenesis needs to be clarified, it seems quite logical that it occurs by regulating germ cell apoptosis, because mRHBDD1 knockdown GC-1 cells were more sensitive to apoptotic stimuli than negative control cells. An apparently indispensable role for mRHBDD1 during this process has been demonstrated by the present *in vivo *results. This warrants additional studies to determine whether malfunction of this enzyme may be a basis of male infertility. This information may be useful for the development of a male contraceptive.

## Conclusion

The mRHBDD1 gene may be associated with the regulation of mammalian spermatogenesis by regulating apoptosis in spermatogonia.

## Methods

### Cell culture and transfection

GC-1 cells, a mouse-derived spermatogonia line, were purchased from ATCC (ATCC number: CRL-2053) and cultured in fresh Dulbecco's modified Eagle's medium supplemented with 10% foetal bovine serum in 5% CO_2 _incubators at 37°C. Adherent cells were passaged every 2–3 days with 0.5 mg/ml trypsin (1:250) and 0.53 mM EDTA. All transfections were performed using Lipofectamin 2000 (Invitrogen).

### Plasmid construction

An RNAi targeting sequence against *mrhbdd1 *(mouse homolog of *rhbdd1*) designated 6# (sense primer: GATCCGAATCAGCCTGACTTCAAATTCAAGAGATTTGAAGTCAGGCTGATTCTTTTTTA; antisense primer: AGCTTAAAAAAGAATCAGCCTGACTTCAAATCTCTTGAATTTGAAGTCAGGCTGATTCG) was cloned into pRNAT-H1.1/Hygro.

### RT-PCR

Male BALB/c mice and Sprague-Dawley rats were obtained from the Laboratory Animal Centre of PUMC (Beijing, China). The Animal Ethics Committee of the National Research Institute for Family Planning Beijing approved the animal experimentation protocols and all animal experiments were performed according to the Guidelines for the Care and Use of Laboratory Animals established by the Chinese Council on Animal Care. The mice and rats were anesthetized under ether. The following organs were excised: intestine, muscle, spleen, liver, lung, heart, kidney, brain, epididymis, stomach and testis. The tissues were immediately immersed in liquid nitrogen and stored at -80°C pending use. Trizol reagent (Invitrogen) was used to isolate total RNA following the manufacturer's instructions. A SuperScript™ First-Strand Synthesis System (Invitrogen) was used for RT-PCR following the manufacturer's instructions.

### Western blotting

Thirty μg of protein was loaded on each lane of a 12% SDS-polyacrylamide gel, separated and then electrophoretically transferred on to a PVDF membrane (GE Healthcare). The membrane was blocked in 5% skim milk for 1 h at room temperature and then incubated with polyclonal rabbit anti-RHBDD1 (Sigma), anti-HA (Sigma), anti-GFP (Santa Cruz), anti-caspase 3 (Santa Cruz) or anti-β-actin (Santa Cruz) overnight at 4°C. The membrane was incubated with anti-rabbit or anti-mouse HRP-IgG (Santa Cruz) for 1 h at room temperature. Chemiluminescence was detected using an ECL blot detection system (Santa Cruz).

### Induction of apoptosis in GC-1 cells

For UV-induced apoptosis, mRHBDD1 knockdown or negative control stable GC-1 cells were exposed to UV irradiation (65 mJ). The medium was changed and cells were harvested 4, 5, 6, 7, 8 or 12 h post UV treatment. For PS341-induced apoptosis, cells were treated with 1 μM PS341 and were harvested 12 h post treatment.

### Flow cytometry

Cells were removed from the plates using 0.25% trypsin-EDTA at 37°C for 5 min, collected, and fixed with 75% ethanol for 30 min at 4°C. They were stained with 50 μg/ml propidium iodide and 100 μg/ml RNase A in PBS at 37°C for 20 min. The DNA content of 10,000 cells was analyzed using a COULTER flow cytometer (EPICS-XL) with EXPO32-ADC software. The percentage of apoptotic cells (% of total cells) was analysed by the program EXPO32-ADC and shown as a bar chart (Microsoft Excel).

### Cell proliferation assay

Cells were seeded at a density of 1000/well in 96-well plates in DMEM with 10% FBS. The medium was changed regularly, and measurements of proliferating cells were performed once per day for 5 days. For the MTS assay (Promega), 20 μl of a combined MTS/PMS solution was added to each well containing 100 μl culture medium and incubated for 3 h at 37°C, then the absorbance was measured at 490 nm using an ELISA plate reader. For the CCK-8 assay (Dojindo Molecular Technologies), 10 μl of CCK-8 solution was added to each well containing 100 μl culture medium and incubated for 3 h at 37°C, then the absorbance was measured at 450 nm using an ELISA plate reader.

### Regeneration of spermatogenesis by GC-1 stable transfectant cells in mouse seminiferous tubules

BALB/c male mice were obtained from the Laboratory Animal Centre of PUMC (Beijing, China). The Animal Ethics Committee of National Research Institute for Family Planning Beijing approved the animal experimentation protocols and all animal experiments were performed according to guidelines (Guidelines for the Care and Use of Laboratory Animals) established by the Chinese Council on Animal Care. The mice were anesthetized under ether. Animals were maintained at 24 ± 1 °C, 55 ± 1% humidity with a 14 h Light: 10 h Dark cycle. Food and water were provided ad libitum. Male mice aged 6–7 weeks were treated with Busulfan (Sigma), 40 mg/kg i.p., to destroy the endogenous germ cells at 4–5 weeks prior to the transplantation. A recipient mouse was anesthetized and the testis was exteriorized through a midline abdominal incision. A sample of approximately 10 μL of cell suspension was injected into the recipient mouse testis by the rete testis, whereby approximately 60%–90% of the surface tubules were filled with cells. For each recipient mouse, one testis was used as the test site for the transplanted stably expressed pRNAT/H1.1/Hygro-negative and the contralateral testis was used for transplantation of the stably expressed pRNAT-H1.1/Hygro-6#.

At 8 weeks after transplantation, the treated and control testes were excised for histological examination. The testis tissues were embedded in O.C.T compound, snap-frozen in liquid nitrogen and cryosectioned. Forty sections were prepared from each testis and examined by fluorescent microscopy (Microphot Nikon) to identify the donor-derived germ cells. Sections were selected, fixed with 4% paraformaldehyde in PBS, stained with TRITC/Rhodamine conjugated peanut agglutinin (PNA) (Sigma) to visualize acrosomes, followed by staining with Hoechst 33258 (Sigma) to visualize nuclei and re-examined by confocal laser microscopy (Leica, Germany).

In our experiment, approximately 80% of the surface tubules were filled with cells in the control and test testis.

### Statistical analysis

The results are expressed as means ± SD. Statistical comparisons used Student's t-test. P < 0.05 was considered statistically significant.

## Authors' contributions

YW, WS, SL and XG carried out all experiments. SZ observed and analyzed the slides. YW and SSK wrote and revised the manuscript. YW, SM and LW conceived of the study and participated in its design and coordination. All authors read and approved the final manuscript.
